# Treatment-induced anaemia and its potential clinical impact in patients receiving sequential high dose chemotherapy for metastatic testicular cancer

**DOI:** 10.1038/sj.bjc.6600629

**Published:** 2002-11-04

**Authors:** C Bokemeyer, K Oechsle, J T Hartmann, P Schöffski, N Schleucher, B Metzner, J Schleicher, L Kanz

**Affiliations:** Department of Haematology/Oncology, University of Tuebingen Medical Centre, Otfried-Müller Str.10, 72076 Tuebingen, Germany; Department of Haematology/Oncology, Hannover Medical School, Carl-Neuberg-Str. 1, 30625 Hannover, Germany; Department of Haematology/Oncology, University of Essen, Hufelandstr. 55, 45122 Essen, Germany; Department of Haematology/Oncology, City Hospital Oldenburg, Dr. Eden-Str. 10, 26133 Oldenburg, Germany; Department of Oncology, Katharinenhospital Stuttgart, Kriegsbergstr. 60, 70174 Stuttgart, Germany

**Keywords:** germ cell tumour, anaemia, prognostic factors, autologous blood stem cell transplantation, chemotherapy, cisplatin

## Abstract

First-line sequential high dose chemotherapy is under investigation in patients with ‘poor prognosis’ metastatic germ cell tumours in order to improve survival. Despite the use of autologous peripheral blood stem cell transplantation and granulocyte colony stimulating factor chemotherapy dose intensification is associated with severe haematotoxicity including anaemia, which may significantly affect quality of life and tolerability of chemotherapy. This study investigates the frequency and degree of anaemia in patients receiving first-line sequential high dose chemotherapy for metastatic testicular cancer and the impact of anaemia on treatment outcome. A total of 101 newly diagnosed patients with ‘poor prognosis’ metastatic nonseminomatous germ cell tumours were treated with one cycle of standard VIP followed by three cycles of HD-VIP-chemotherapy (etoposide, ifosfamide, cisplatin) within a large phase I/II study. Differential blood cell counts were taken prior, during and after every cycle of chemotherapy. Additionally, the numbers of red blood cell and platelet transfusions were recorded. Kaplan–Meier analyses were performed to correlate pre-treatment and post-treatment haemoglobin values to response and overall survival. Forty-eight per cent of the patients were classified anaemic (haemoglobin <12 g dl^−1^) prior to the start of chemotherapy. The application of sequential HD-VIP resulted in median haemoglobin nadirs between 7.8 g dl^−1^ (range 5.5–11.1 g dl^−1^) in the first cycle and 7.6 g dl^−1^ (range 6.0–11.4 g dl^−1^) in the third cycle despite the frequent use of red blood cell transfusions. Almost all patients (99%) had haemoglobin levels <10 g dl^−1^ at some timepoint during first-line sequential high dose chemotherapy. Overall, 97 patients received red blood cell transfusions with a median of 10 units (range 2–25) per patient during the four consecutive cycles of therapy. The time to first transfusion was shortest in patients with the lowest initial haemoglobin values. While there was no prediction of response or outcome by baseline haemoglobin-levels, a significant survival difference in favour of patients with a haemoglobin value >10.5 g dl^−1^ after completion of four cycles of therapy (at leukocyte recovery after the last cycle) compared to those with haemoglobin values <10.5 g dl^−1^ was found with 3-year overall survival rates of 87% *vs* 68%, respectively (*P*<0.05). Severe anaemia is a very frequent side effect of sequential dose intensive therapy in patients with germ cell cancer, with almost all patients becoming transfusion dependent. Despite the frequent use of red blood cell transfusions, median haemoglobin nadirs remained about 7.5–8 g dl^−1^ during therapy. A correlation of haemoglobin-values after completion of therapy to overall treatment outcome was found.

*British Journal of Cancer* (2002) **87**, 1066–1071. doi:10.1038/sj.bjc.6600629 www.bjcancer.com

© 2002 Cancer Research UK

## 

With the use of cisplatin-based combination chemotherapy, 70–90% of the patients with metastatic testicular cancer will be cured (Bokemeyer *et al*, 1998; Hartmann *et al*, 1999). However, in patients fulfilling ‘poor prognosis’-criteria according to the IGCCCG classification the actual 5-year survival rate is only 50–60% (Igcccg, (1997); Sonneveld *et al*, 2001). A matched pair analysis has revealed a survival benefit of 15–20% for patients treated with sequential first-line high dose chemotherapy (HD-Ctx) plus autologous peripheral blood stem cell transplantation (PBSCT) compared to patients receiving standard dose chemotherapy (Bokemeyer *et al*, 1999). Randomised studies comparing HD-Ctx to cisplatin-based standard dose chemotherapy are currently ongoing.

The intensification of first-line treatment using the dose escalation of etoposide and ifosfamide is associated with a certain degree of acute toxicities, particularly gastrointestinal side effects and haematotoxicity. Intensive sequential HD-chemotherapy is associated with the risk of severe neutropenia, thrombocytopenia and anaemia despite the use of PBSC retransplantation and haematopoetic growth factors (granulocyte colony stimulating factor) (G-CSF). There is an increasing awareness among the oncology community that the decrease of haemoglobin (Hb) concentrations may lead to physical and psychological consequences that adversely effect the quality of life of cancer patients (Eguchi, 1995; Thatcher, 1998; Groopman and Itri, 1999; Ray-Coquard *et al*, 1999; Barrett-Lee *et al*, 2000; Mercadante *et al*, 2000; Littlewood *et al*, 2001).

The cumulative risk of receiving red blood cell (RBC) transfusions in patients who are treated with 4–6 cycles of conventional cisplatin-based combination chemotherapy is approximately 60% (Manegold, 1998; Barrett-Lee *et al*, 2000; Pivot *et al*, 2000). RBC transfusions may be associated with adverse effects such as viral infections, e.g. hepatitis and HIV, bacterial and parasitic infections, haemolysis and immunologic or anaphylactic reactions (Eguchi, 1995; Thatcher, 1998; Groopman and Itri, 1999; Ray-Coquard *et al*, 1999; Mercadante *et al*, 2000).

The aim of this analysis was to describe the frequency and degree of anaemia in patients undergoing first-line sequential HD-chemotherapy for ‘poor prognosis’ metastatic germ cell tumours. Additionally, the relation between haemoglobin concentrations and treatment outcome has been investigated.

## PATIENTS AND METHODS

This study has analysed patients with metastatic testicular germ cell tumours (GCT) undergoing first-line high dose chemotherapy between 1997 and 1999. All patients were treated within a multicentre phase I/II study of the German Testicular Cancer Study Group in collaboration with EORTC GU-Group treatment centres investigating sequential HD-VIP (etoposide, ifosfamide, cisplatin) therapy with PBSCT as first-line treatment in patients with ‘poor prognosis’ GCT according to IGCCCG criteria (Bokemeyer *et al*, 1999).

Previously untreated male patients with histologically proven nonseminomatous GCT have been included. All patients had to fulfill ‘poor prognosis’ criteria according to the IGCCCG (primary mediastinal tumour or gonadal primary tumour with non-pulmonary visceral metastases or patients with ‘poor markers’ such as AFP >10 000 ng ml^−1^ or β-HCG >50 000 U ml^−1^ or LDH >10 normal value) (Igcccg, (1997)). Patients with impaired renal function (creatinine clearance <50 ml min^−1^), inadequate bone marrow function (leukocytes <3000 μl^−1^ or thrombocytes <100 000 μl^−1^ prior to therapy), other major organ dysfunctions unless caused by malignant disease, evidence of second malignancies except basal cell skin cancer and patients aged over 60 years were excluded. All patients had given written informed consent prior to therapy. The study protocol had been approved by the Ethical Committee of Tuebingen University.

Treatment included one cycle of standard dosed VIP-chemotherapy (cisplatin 20 mg m^−2^ d_1–5_, etoposide 100 mg m^−2^ d_1–5_ and ifosfamide 1.2 g m^−2^ d_1–5_) plus G-CSF (5 μg kg^−1^) to collect autologous (planned separation yield: ⩾6×10^6^ CD34+ cells kg^−1^ bw) followed by 3 cycles of HD-VIP chemotherapy, consisting of cisplatin 20 mg m^−2^ d_1–5_, etoposide 300 mg m^−2^ d_1–5_ and ifosfamide 2000 mg m^−2^ d_1–5_ (level 6) or 2400 mg m^−2^ (level 7) every 3 weeks given with reinfusion of PBSC at day 7 and G-CSF support from day 7 until neutrophil recovery >1000 μl^−1^ for 2 consecutive days (Table 1

**Table 1:**
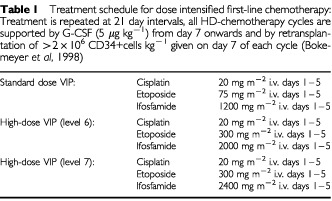
Treatment schedule for dose intensified first-line chemotherapy: Treatment is repeated at 21 day intervals, all HD-chemotherapy cycles are supported by G-CSF (5 μg kg^−1^) from day 7 onwards and by retransplantation of >2×10^6^ CD34+cells kg^−1^ given on day 7 of each cycle (Bokemeyer *et al*, 1998

). This regimen has been previously reported (Bokemeyer *et al*, 1999). Only 101 patients who received all four cycles of chemotherapy were included in this retrospective evaluation.

Differential blood cell counts were taken prior to the start of therapy, at the beginning of every new cycle of therapy and twice weekly during chemotherapy until white blood cell counts and platelet levels had recovered to >2000 and >50 000 μl^−1^, respectively. Anaemia was defined as a Hb level <12 g dl^−1^. RBC transfusions were recommended to be used in patients with a decrease of Hb levels below 8 g dl^−1^ or in case of clinical symptoms of anaemia. When available at the participating centres, patients received filtered transfusions of erythrocytes to reduce the transfusion of contaminated white blood cells to a minimum. The number and days of RBC transfusions were recorded during each cycle. Additionally, the number of patients receiving platelet transfusions–recommended threshold of platelet counts <10 000 μl^−1^ or signs of bleeding–were registered.

Response to therapy was evaluated by computed scans of lungs, abdomen and–in case of cerebral metastases–of the brain as well as by measurement of serum tumour marker concentrations of human chorionic gonadotropin (β-HCG), alfa-fetoprotein (AFP) and lactate dehydrogenase (LDH). Tumour status assessments were performed prior to therapy, after the second cycle and at the end of therapy, and the results were classified according to WHO criteria. To compare the development of anaemia to response to therapy and to overall survival time, the patients were divided into separate groups according to the Hb level prior to the start of treatment and according to the Hb nadir after therapy. Treatment results for the groups of patients either above or below the median Hb-value of the total group were compared. The chi-square test was employed for group comparisons. Analyses on long term outcome were performed using the Kaplan–Meier method with logrank test for the groups of patients with different degrees of anaemia.

## RESULTS

One hundred and one patients with ‘poor prognosis’ metastatic GCT treated with HD-VIP chemotherapy have been retrospectively analysed with respect to anaemia and treatment outcome. Patient characteristics are given in Table 2

**Table 2:**
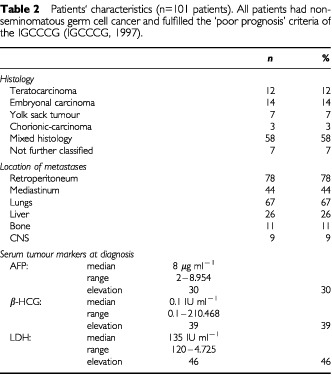
Patients` characteristics (n=101 patients). All patients had nonseminomatous germ cell cancer and fulfilled the ‘poor prognosis’ criteria of the IGCCCG (IGCCCG, 1997)

. The median age of the patients was 30 years. The median follow-up duration since start of chemotherapy was 41 months (range 5–78). All patients alive had a minimum follow-up of 1 year.

The median Hb concentration prior to start of chemotherapy was 12.2 g dl^−1^ (range 7.5–17.4). Fifty-three patients (52%) had Hb levels within the normal range (⩾12 g dl^−1^) and 48 patients (48%) started treatment with Hb levels <12 g dl^−1^. Two patients without any detectable bleeding presented with Hb levels <8 g dl^−1^ at diagnosis and received RBC transfusions prior to the start of chemotherapy. There was no microscopically detectable bone marrow involvement by germ cell tumour cells in these two patients (Table 3

**Table 3:**
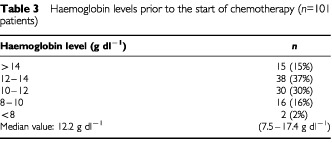
Haemoglobin levels prior to the start of chemotherapy (*n*=101 patients)

).

After completion of one cycle of standard dose and three cycles of HD-VIP chemotherapy the median Hb level dropped to 10.4 g dl^−1^ (range 8.2–14.5). Overall, only three patients (3%) did not require any RBC transfusion during the total treatment sequence. The remaining 98 patients (97%) have been transfused receiving a median of 10 RBC units (range 2–25). The median Hb decrease during all treatment cycles was 2.3 g dl^−1^ (range 0–7.8). There was no significant difference in the degree of anaemia between patients receiving the two different chemotherapy dose levels 6 and 7 (total ifosfamide doses 10 or 12 g m^−2^ cycle^−1^, respectively).

Following the standard dose VIP induction cycle, the median Hb nadir was 9.6 g dl^−1^ (range 6.1–15.8). Only 11 patients did not become anaemic (Hb <12 g dl^−1^) during the initial cycle of standard dose chemotherapy and another 27 patients (27%) remained at a Hb level >10 g dl^−1^. Twenty-seven per cent of the patients were transfused during the first cycle (median number of units 2; range 2–8).

The application of the first cycle of HD-VIP resulted in a median Hb nadir of 7.8 g dl^−1^ (range 5.5–11.1). This corresponded to a median Hb decrease of 2.8 g dl^−1^. Three patients (3%) preserved Hb levels >10 g dl^−1^, none of the patients remained at Hb values >12 g dl^−1^. Seventy-six patients (76%) were transfused, receiving a median of 4 RBC units (range 2–9). After administration of the second cycle of HD-VIP Hb levels dropped to 7.8 g dl^−1^ and 85% of the patients required a median of 4 units of RBC transfusions (range 2–12). The median Hb nadir during the third cycle of HD-VIP was 7.6 g dl^−1^. In this cycle, 91% of the patients (*n*=91) required a median of 6 units of RBC (range 2–13). During the second and the third cycle of HD-VIP only one patient (1%) remained at a Hb level >10 g dl^−1^. In almost all patients (99%) Hb levels declined to values <10 g dl^−1^ (Table 4

**Table 4:**
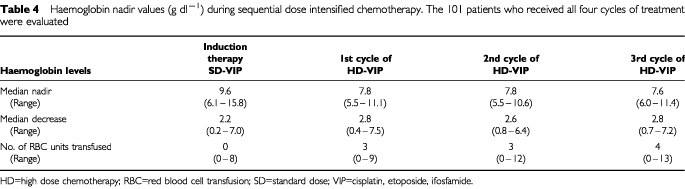
Haemoglobin nadir values (g dl^−1^) during sequential dose intensified chemotherapy. The 101 patients who received all four cycles of treatment were evaluated

).

The correlation of the median Hb level prior to chemotherapy of the 98 transfused patients to the time until first transfusion showed a median Hb level of 10.4 g dl^−1^ at start (range 7.7–17.4) in the 27 patients receiving RBC transfusion during the first cycle of standard chemotherapy compared to 13.9 g dl^−1^ (range 9.8–16.5) in six patients receiving their first RBC transfusion in the third cycle of high dose chemotherapy. Fifty-four patients were transfused for the first time during the first cycle of high dose VIP with a median Hb level of 11.9 g dl^−1^ (range 9.9–17.4) prior to the start of therapy. During the second cycle of HD-VIP 11 patients received their first RBC transfusion, having started chemotherapy with a Hb level of 12.7 g dl^−1^ (range 7.5–15.7).

Patient characteristics with respect to sites of metastases and chemotherapy dose levels (6 *vs* 7) were not significantly different among those groups

Forty-two of 56 patients (75%) starting chemotherapy with Hb-levels ⩾10.5 g dl^−1^ and 35 of 45 patients (77%) with Hb levels below 10.5 g dl^−1^ achieved a complete or marker negative partial remission (n.s.). Analysis of survival times was performed using the Kaplan–Meier method revealing similar 3-year-progression free survival rates in both groups: 76% for the group of patients with a pretreatment Hb value ⩾10.5 g dl^−1^ *vs* 68% for patients with Hb levels <10.5 g dl^−1^ (n.s.).

**Figure 1:**
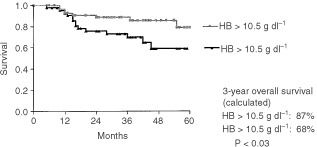
Overall survival time of patients with Hb values > or <10.5 g dl^−1^ calculated since completion of the HD-VIP-chemotherapy.

Patients with low Hb-levels (<10.5 g dl^−1^) after completion of HD-VIP (defined as timepoint at 2–3 weeks after last CT-application but at full neutrophil recovery) had achieved a response rate of 70%, whereas in the group of patients with higher Hb values after treatment (>10.5 g dl^−1^) the response rate was determined as 85% (*P*=0.07). After a median follow up of 41 months since diagnosis the calculated 3-year overall survival rates among both groups were significantly different (87% for the group with higher Hb after therapy *vs* 68% for patients with a lower Hb value after therapy, 95% confidence interval 0.1692–0.9335, *P*<0.03) (Figure 1

). The distribution of the patients tumour characteristics and median Hb-nadirs during therapy for these two groups are given in Table 5

**Table 5:**
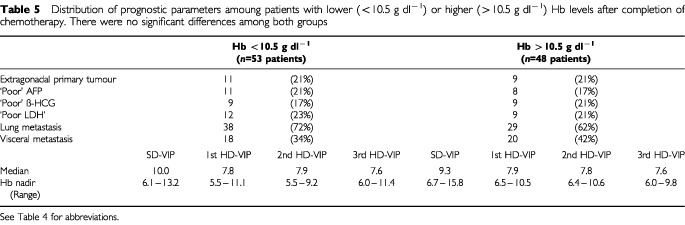
Distribution of prognostic parameters amoung patients with lower (<10.5 g dl^−1^) or higher (>10.5 g dl^−1^) Hb levels after completion of chemotherapy. There were no significant differences among both groups

. The were no significant differences.

The degree of thrombocytopenia during intensified chemotherapy – representing another parameter describing the degree of haematotoxicity associated with the HD-VIP regimen – was also analysed (Table 6

**Table 6:**
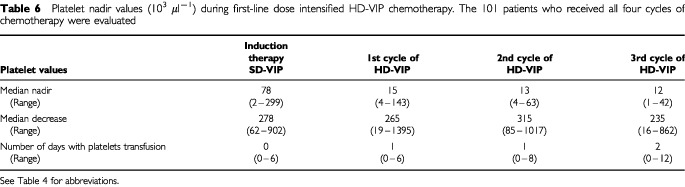
Platelet nadir values (10^3^ μl^−1^) during first-line dose intensified HD-VIP chemotherapy. The 101 patients who received all four cycles of chemotherapy were evaluated

). After application of the standard dose VIP chemotherapy the median platelet nadir was 78 000 μl^−1^ (range 9000–299 000 μl^−1^). During the three cycles of high dose VIP chemotherapy the median platelet nadir was 12 000 μl^−1^ (range 1000–143 000 μl^−1^). While most patients (85%) received no platelet transfusions (range 0–6) during the standard dose VIP cycle, patients were transfused at a median of 2 days (range 0–12) during each high dose cycle. The number of patients receiving platelet transfusions increased from 15 (15%) during standard VIP to 97 (96%) during high dose chemotherapy.

## DISCUSSION

This report describes the frequency and severity of anaemia in patients receiving cisplatin-based first-line sequential high dose chemotherapy (HD-VIP) plus autologous PBSC transplantation for the treatment of ‘poor prognosis’ metastatic testicular cancer. Taking into account strict criteria for the definition of anaemia (<12 g dl^−1^) already 50% of these young male patients with advanced metastatic germ cell cancer have been anaemic at diagnosis. During the first cycle of VIP standard dose chemotherapy the incidence of anaemia (Hb <12 g dl^-1^) reached 90% and about a quarter of patients dropped to Hb levels <10 g dl^−1^, indicating the impact of standard dose cisplatin based chemotherapy for the induction of anaemia. Summarizing data from all cycles of HD-VIP chemotherapy the number of patients with Hb level <10 g dl^−1^ was 99% and the median Hb nadir value was 7.8 g dl^−1^ demonstrating the significant degree of anaemia associated with this dose intensified chemotherapy approach despite the frequent use of RBC transfusions. The frequency of RBC transfusions increased from 27% during the standard dose VIP cycle to 91% during HD-VIP. The patients who were transfused, received a median number of 5 units of RBC transfusions in association with each HD-VIP cycle. During the whole treatment period 97% of all patients required RBC transfusions with a median total number of 10 RBC packs applied. The time to first transfusion was correlated to the Hb level prior to the start of chemotherapy with a median Hb value of 10.4 g dl^−1^ for patients being transfused in the first cycle compared to 13.9 g dl^−1^ for patients who received their first RBC transfusion during the last cycle. These findings indicate the important prognostic value of the pre-treatment Hb level for the risk of developing transfusion dependent anaemia, as reported by other investigators (Manegold, 1998; Groopman and Itri, 1999; Barrett-Lee *et al*, 2000). With the higher number of transfusions given, the potential side effects of RBC transfusions, such as viral infections, haemolysis, immunological or anaphylactic reactions may contribute to the risk associated with the treatment in this young patient population (Groopman and Itri, 1999; Mercadante *et al*, 2000). This is of particular importance, since between 50% and 80% of all patients will be cured from testicular cancer by this dose intensified treatment approach (Bokemeyer *et al*, 1998; Sonneveld *et al*, 2001). With a median of 10 RBC units transfused to each patient, the impact of cisplatin-based sequential high dose chemotherapy on erythropoiesis and the importance of therapy-induced anaemia is clearly demonstrated. It must be kept in mind that the RBC transfusion approach was conservative, since the common policy to transfuse only the amount of RBC packages absolutely necessary has led to decreased Hb concentrations in most patients keeping them in the range between 8 and 10 g dl^−1^ during the total treatment period.

Recent studies in ovarian or lung cancer patients receiving cisplatin-based chemotherapy have demonstrated that higher Hb levels will exert a positive effect on the patients' tolerability of chemotherapy (Manegold, 1998; Obermair *et al*, 1998; Pivot *et al*, 2000). Patients with low Hb-levels due to the disease itself or due to myelotoxic chemotherapy have a worse physical and psychological constitution and a lower capacity to compensate for treatment toxicity. Recently, two prospective studies in oncological patients have demonstrated a significant effect of Hb concentrations during chemotherapy on quality of life (Gabrilove *et al*, 2001; Littlewood *et al*, 2001).

With their potential side effects RBC transfusions are not the optimal solution to treat anaemia in patients undergoing chemotherapy. The prophylactic application of recombinant human erythropoietin (rHu-EPO) is capable of preventing anaemia and to decrease the frequency of transfusions in patients receiving cisplatin-based and non-platinum containing chemotherapy in several clinical trials (Henry, 1998; Ludwig and Fritz, 1998; Thatcher, 1999; Adamson and Ludwig, 1999; Crawford *et al*, 1999; Ponchio *et al*, 2000).

Recent studies have also shown a trend for improved survival associated with higher Hb-levels due to rHu-EPO application in solid tumour patients receiving non-platinum based chemotherapy (Gabrilove *et al*, 2001; Littlewood *et al*, 2001). Higher Hb levels during treatment might be beneficial in terms of chemotherapy response and improved treatment outcome. Another trial demonstrated a correlation between Hb levels during chemotherapy and response to therapy. This investigation has included patients with different types of solid tumours (Barrett-Lee *et al*, 2000).

In our study no correlation between the initial Hb level prior to the start of treatment and response to and outcome of therapy could be demonstrated, which might be explained by the relative mild degree of anaemia initially present in the analysed cohort. On the other hand a significant correlation between therapy outcome and Hb values after completion of chemotherapy was found in this study. This significant correlation translated into a long-term survival benefit for the group of patients with higher Hb-levels (87% *vs* 68% 3-year overall survival). This finding was not explained by different clinical characteristics of the patients included into the two groups as shown in Table 5. Although the present investigation is limited due to its retrospective character these findings may stimulate further research into the association between anaemia and outcome of chemotherapy. Interactions may be possible on several levels such as, e.g., increased bloodflow and drug delivery to the tumour in less anaemic patients or higher effectiveness of radical generating agents in the presence of better oxygen supply as postulated for radiotherapy (Haensgen *et al*, 2001). Since anaemia is a major problem in dose intensive therapy in the young patient population with germ cell cancer the effects of erythropoietic growth factors on the frequency of transfusions, quality of life and potentially also on overall response to chemotherapy should be further studied.
